# Un cas rare d'hémangiopéricytome malin du membre inférieur

**DOI:** 10.11604/pamj.2014.19.19.4377

**Published:** 2014-09-09

**Authors:** Zouhir Ameziane Hassani, Abdelkrim Rhanim

**Affiliations:** 1 Service de Traumatologie Orthopédie, Hôpital Avicenne, CHU Ibn Sina, Université Mohammed V-Souissi Rabat, Maroc

**Keywords:** Tumeur vasculaire, hémangiopericytome, sarcome, scintigraphie osseuse, vascular tumor, hemangiopericytoma, sarcoma, bone scan

## Image en medicine

L'hémangiopericytome est une tumeur vasculaire rare classée comme sarcome des tissus mous, elle peut être bénigne ou maligne, elle prend naissance au niveau des péricytes qui sont des cellules localisées au niveau de la lame basale de l'endothélium des capillaires ce qui explique son caractère ubiquitaire. Leur traitement de référence est la chirurgie souvent complétée d'une radiothérapie. Nous rapportons un cas d'un énorme hémangiopericytome malin du pied chez une patiente de 28 ans qui nous a été adressé pour une tumeur du pied gauche nécrosée est infectée avec une biopsie qui a été réalisée revenant en faveur d'un hémangiopericytome le bilan d'extension comportant une scintigraphie osseuse et un scanner thoraco-abdomino-pelvien n'a pas objectivé de localisation secondaire Vu la taille de la tumeur et son caractère nécrosé et infectée un traitement chirurgical radical par une amputation transtibiale a été décidé après le consentement de la patiente.

**Figure 1:**
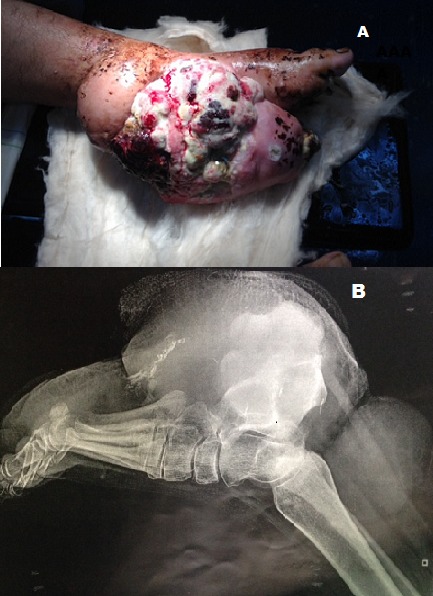
(A): hémangiopericytome malin du pied; (B): radiographie standard montrant l'extension de la tumeur vers le calcanéum

